# MoO_3_/WO_3_/rGO as electrode material for supercapacitor and catalyst for methanol and ethanol electrooxidation

**DOI:** 10.1038/s41598-024-59018-2

**Published:** 2024-04-30

**Authors:** Mohammad Bagher Askari, Parisa Salarizadeh, Mohammad Hassan Ramezan zadeh

**Affiliations:** 1 Department of Semiconductor, Institute of Science and High Technology and Environmental Sciences, Graduate University of Advanced Technology, Kerman, Iran; 2 High-Temperature Fuel Cell Research Department, Vali-e-Asr University of Rafsanjan, Rafsanjan, Iran; 3 Department of Electrical-Electronics Engineering, Abdullah Gül University, 38080 Kayseri, Turkey

**Keywords:** MoO_3_/WO_3_/rGO, Supercapacitor, Methanol oxidation reaction, Ethanol oxidation reaction, Analytical chemistry, Electrochemistry, Energy

## Abstract

The potential of metal oxides in electrochemical energy storage encouraged our research team to synthesize molybdenum oxide/tungsten oxide nanocomposites (MoO_3_/WO_3_) and their hybrid with reduced graphene oxide (rGO), in the form of MoO_3_/WO_3_/rGO as a substrate with relatively good electrical conductivity and suitable electrochemical active surface. In this context, we presented the electrochemical behavior of these nanocomposites as an electrode for supercapacitors and as a catalyst in the oxidation process of methanol/ethanol. Our engineered samples were characterized by X-ray diffraction pattern and scanning electron microscopy. As a result, MoO_3_/WO_3_ and MoO_3_/WO_3_/rGO indicated specific capacitances of 452 and 583 F/g and stability of 88.9% and 92.6% after 2000 consecutive GCD cycles, respectively. Also, MoO_3_/WO_3_ and MoO_3_/WO_3_/rGO nanocatalysts showed oxidation current densities of 117 and 170 mA/cm^2^ at scan rate of 50 mV/s, and stability of 71 and 89%, respectively in chronoamperometry analysis, in the MOR process. Interestingly, in the ethanol oxidation process, corresponding oxidation current densities of 42 and 106 mA/cm^2^ and stability values of 70 and 82% were achieved. MoO_3_/WO_3_ and MoO_3_/WO_3_/rGO can be attractive options paving the way for prospective alcohol-based fuel cells.

## Introduction

The lack of fossil fuel resources, which are currently the main sources of energy production, will soon face the world with an energy crisis^1^. The policies of different governments and countries are different towards this crisis, but almost all countries, either developed or developing ones, have come to the conclusion that the use of renewable fuel sources is a necessity^2^. Almost all countries in the world, depending on their geographical location, can benefit from resources such as sunlight, tides of the sea, wind, geothermal energy, etc^3^.

Having renewable resources is a reason for developing clean energy; moreover, the design and construction of modern energy production and storage equipment that utilizes these clean fuel sources are considered one of the most important and practical sciences around the world^4^. Various types of solar cells, geothermal facilities, and wind turbines are some examples of energy conversion equipment^5^. With the development of nanoscience, materials, and electrochemistry, recently, ultramodern and alluring equipment such as fuel cells, batteries, and electrochemical supercapacitors have received much attention. Almost every day we see the introduction of advanced, highly efficient materials in the field of electrode materials suggested for utilization in supercapacitors and fuel cell catalysts.

In general, the advantage of electrode materials is that they are inexpensive and highly efficient in the field of energy storage such as electrochemical batteries and supercapacitors^6,7^, while the catalysts used in the anode and cathode structures of alcohol fuel cells are generally expensive including rare materials such as platinum, ruthenium, palladium, etc^8^.

Finding a material with common use both as an electrode material in a supercapacitor and as a catalyst in an alcohol fuel cell can lead to very interesting research. In the field of materials application in supercapacitor electrodes, a general classification can be mentioned, by distinguishing between materials with a capacitive behavior, like various types of carbons^9^, giving rise to electric double layer capacitors (EDLCs), and materials with a redox peak in their CV curves, corresponding to pseudocapacitors or battery-type devices^10,11^.

Examining the state-of-the-art sources in the field of advanced electrodes capable of being used in supercapacitors shows that metal oxides are the most widely used materials and at the same time the most efficient materials in this field^12^. Supplementarily, the use of metal oxides as a catalyst has also recently received much attention. The green and environmentally friendly synthesis, the cheap precursors, the cyclic stability, and the relatively low toxicity have led researchers to apply these materials in various fields of electrochemistry^13–15^.

Tungsten oxide and molybdenum oxide are among the most widely used metal oxides in catalyst science^16–18^. These materials have been used in single, composite, and hybrid forms, as catalysts for sensing, and detecting various drugs and substances, and for energy storage and production^19–22^. However, it seems that the composite consisting of MoO_3_ and WO_3_ has not been explored as a catalyst in both alcohol fuel cells and electrodes as supercapacitors. Transition mixed metal oxides have several advantages over single metal oxides. For example, different oxidation states make them catalytically active.

However, due to the different catalytic and storage behaviors of various oxides, the use of mixed metal oxides could offer some interesting advantages.

The advantages of alcohol fuel cells, such as operation at low temperatures, high energy density, small dimensions, compatibility with the environment, and the production of water as the final product^23,24^, encouraged us to synthesize MoO_3_/WO_3_ and MoO_3_/WO_3_/rGO composites. After evaluating the ability of these materials as supercapacitor electrodes, we examined the performance of these materials as catalysts in the oxidation of methanol and ethanol.

Furthermore, we highlighted the positive effect of rGO placement in the MoO_3_/WO_3_ structure showing an increase in the efficiency of the nanocatalyst during the methanol and ethanol oxidation. Similarly, a benefit will be reported in rGO-contained energy storage through increasing the electrochemically active surface area and improving the electrical conductivity.

## Experimental

### Materials and equipment

Ammonium heptamolybdate ((NH_4_)_6_Mo_7_O_24_), sodium tungstate (Na_2_WO_4_), and urea (CH_4_N_2_O) were all purchased from Sigma-Aldrich with a purity of more than 99%. The crystal structure of the material was investigated through X-ray diffraction analysis (XRD, PANalytical X’Pert Pro MRD device), and the surface morphology of the material was done with scanning electron microscopy (SEM, TESCAN VEGA3). Electrochemical tests including cyclic voltammetry (CV), electrochemical impedance spectroscopy (EIS), Chronoamperometry and Galvanostatic charge/discharge (*GCD*) were performed with a Metrohm potentiostat/galvanostat instrument (302N).

### Synthesis and characterization

Compendiously, to synthesize MoO_3_/WO_3_, we dissolved 0.65 g of sodium tungstate, 0.7 g of ammonium heptamolybdate, and 1 g of urea in 60 ml of deionized water using a magnetic stirrer for 30 min. Next, we poured the resulting clear solution into a 100 ml reactor and put it in an oven for 18 h at a temperature of 180 °C. After the reactor cools down, we mixed the obtained product with water and ethanol precipitated in a centrifugation device at 6000 rpm, the process of which is repeated several times each carried out for 6 min. The obtained materials are dried for 8 h in a vacuum oven at a temperature of 50 °C followed by calcination at 400 °C for 2 h. In the second stage, the synthesis of MoO_3_/WO_3_/rGO is done with a similar method as MoO_3_/WO_3_, with the difference that first 0.2 g GO were added to the tungsten, molybdenum, and urea precursors. GO was converted into rGO in a hydrothermal process.

Graphene oxide was synthesized by modified Hummers’ method. 1 g graphite powder was dispersed in a mixture of sulfuric acid and phosphoric acid under magnetic stirring for 1 h. The vessel was put in an ice bath and 9 g KMnO_4_ was slowly added to it. The mixture was stirred for 24 h and after that 100 mL of deionized water was slowly added to it. Then, to eliminate excess KMnO_4_, 35 mL hydrogen peroxide was added and stirred for 10 min. The mixture was centrifuged and the supernatant was decanted. The residual was washed with 0.2 M hydrochloric acid and water and dried in an oven at 90 °C.

### Electrode preparation and modification

All electrochemical measurements are conducted with a three-electrode system for supercapacitor and alcohol oxidation tests. Three electrode systems used in this research included an FTO coated with catalyst (1 cm × 2 cm) as a working electrode, platinum wire, and Ag/AgCl as auxiliary and reference electrodes, respectively. Electrode modification was done by the drop-casting method. The working electrode was modified by the dispersion of 10 mg of MoO_3_/WO_3_ and MoO_3_/WO_3_/rGO in a solution containing 0.5 ml of isopropyl alcohol, 4 µl of Nafion solution 5%, and 1 ml of deionized water for 30 min by ultrasonication. It is e noted that all potentials are versus Ag/AgCl.

## Results and discussion

### Crystal structure and surface morphology of the synthesized nanomaterials

**Figure 1:**
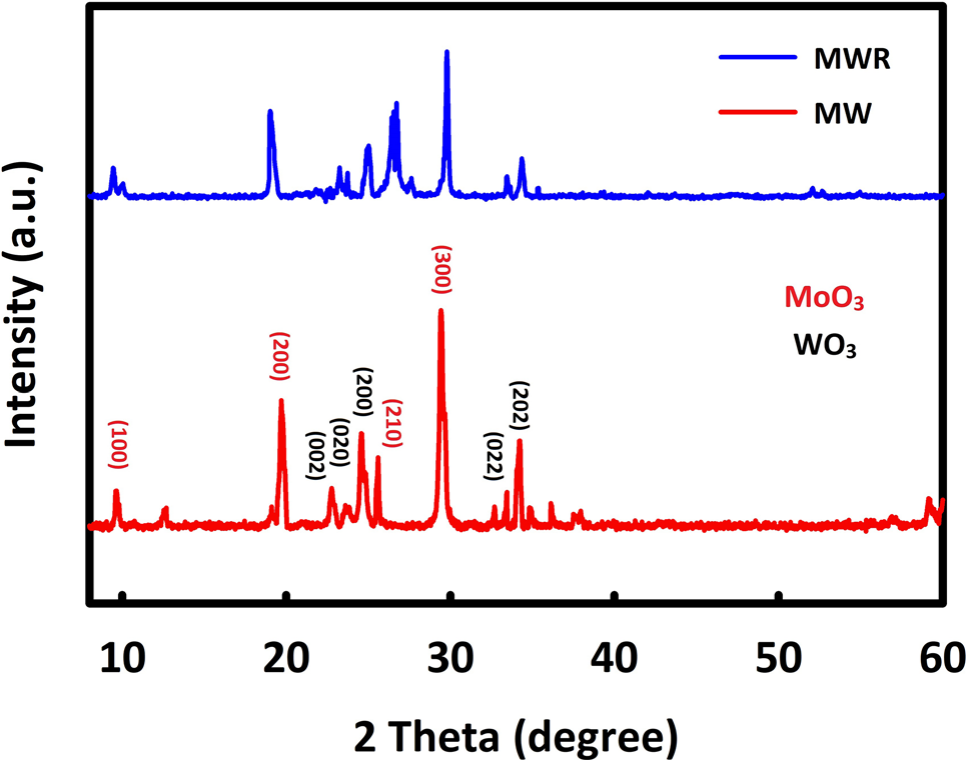
XRD analysis of MoO_3_/WO_3_ (MW) and MoO_3_/WO_3_/rGO (MWR).

The crystal structure of MoO_3_/WO_3_ and MoO_3_/WO_3_/rGO was studied by XRD analysis. MoO_3_ has characteristic peaks at 2θ = 9.7°, 19.3°, 25.6°, and 29.4°, which are indexed as (100), (200), (210), and (300) crystal planes, and this diffraction pattern is compliance consistent with JCPDS Card No. 00-021-0569^25^ and also, the characteristic peaks of WO_3_ in the orthorhombic structure are seen at the diffraction angles of 23.2°, 23.5°, 24.4°, 33.3°, and 34.2°, which correspond to the (002), (020), (200), (022), and (202) planes respectively. This diffraction pattern is also in complete agreement with JCPDS card no.01-072-1465^26^ are shown in Fig. 1. In the X-ray diffraction analysis of MoO_3_/WO_3_/rGO, in addition to the peaks of MoO_3_ and WO_3_, a relatively wide peak at about 26° is seen, which belongs to rGO^27^.

**Figure 2:**
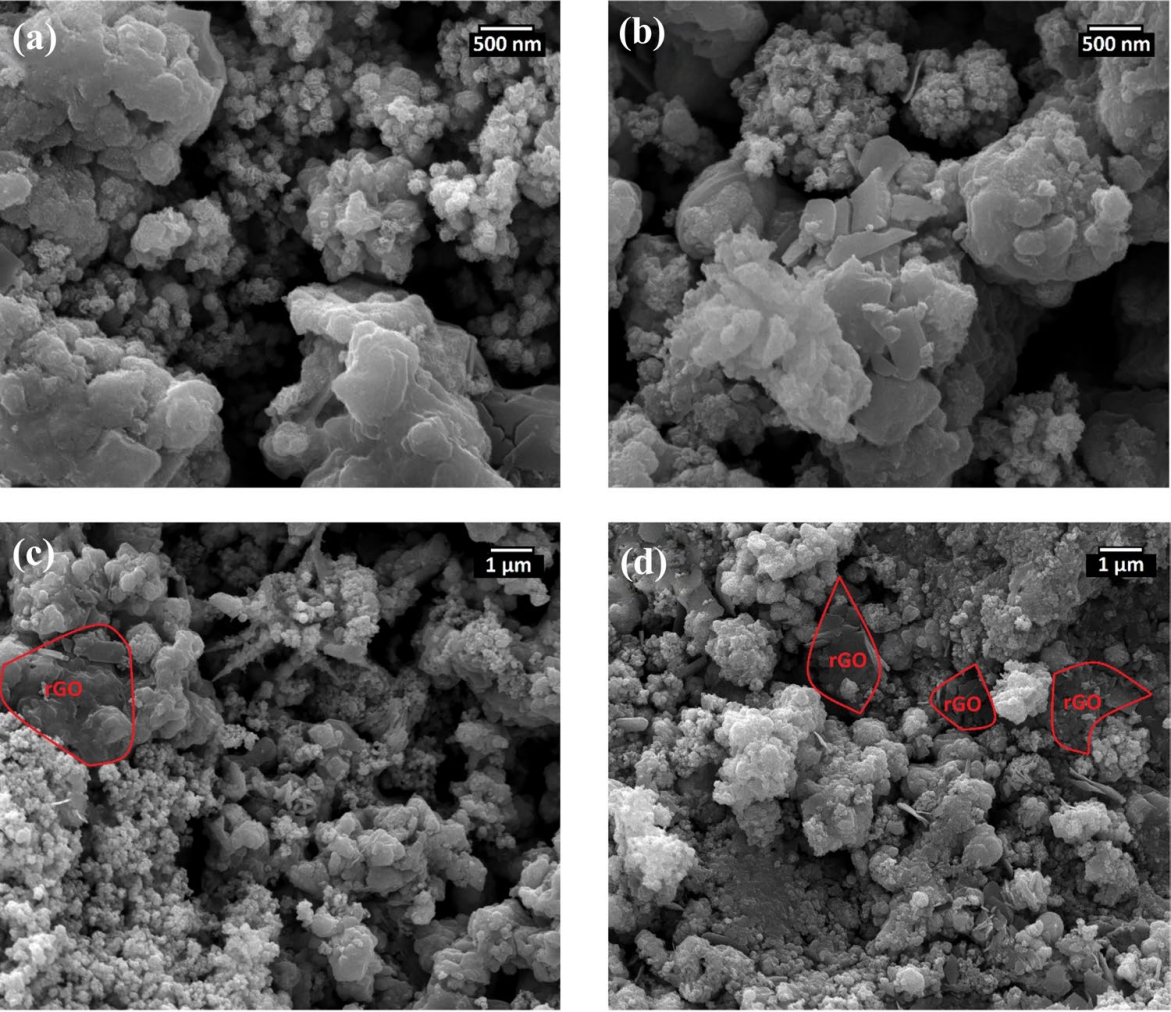
SEM images of MoO_3_/WO_3_ (**a** and **b**), and MoO_3_/WO_3_/rGO (**c** and **d**).

SEM images were acquired to study the surface morphology of MoO_3_/WO_3_ and MoO_3_/WO_3_/rGO nanocatalysts. Figure 2a, b depict the SEM images of MoO_3_/WO_3_ in which the relatively porous surface of this nanocomposite shows off. These pores are crucial in electrochemical processes, in the way that an enhanced electrochemical active surface cause’s easier penetration of the electrolyte into the catalyst core facilitating the electrochemical processes. In Fig. 2c, d, which belongs to MoO_3_/WO_3_/rGO, one can see the uniform incorporation of MoO_3_/WO_3_ on the surface of rGO nanosheets.

Brunauer–Emmett–Teller (BET) analysis was performed to check the porosity and specific surface area of the synthesized nanocomposites. The results of this analysis are shown in Table 1. Results show the higher specific surface area of MoO_3_/WO_3_/rGO than MoO_3_/WO_3_, representing that rGO increased porosity and improved the surface area of the catalyst.

**Table 1:** BET analysis result for MoO_3_/WO_3_ and, MoO_3_/WO_3_/rGO.

Sample	a_s, BET_ [m^2^ g^−1^]	a_s, Lang_ [m^2^ g^−1^]	Mean pore diameter [nm]
MoO_3_/WO_3_	7.5096	107.97	7.5113
MoO_3_/WO_3_/rGO	8.27	186.53	9.019

### Electrochemical studies

#### MoO_3_/WO_3_/rGO and MoO_3_/WO_3_ as supercapacitor electrode

**Figure 3:**
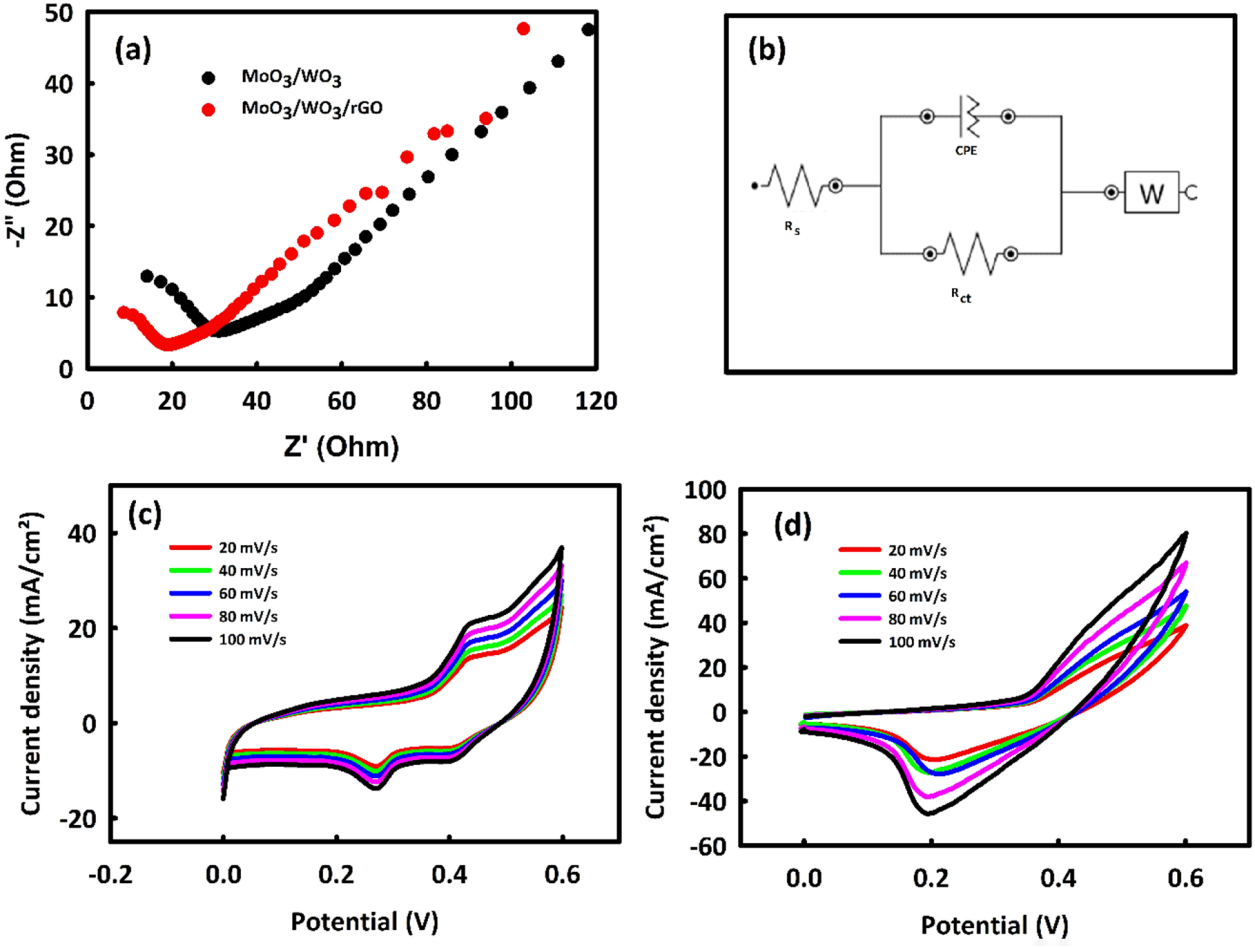
Nyquist plots of MoO_3_/WO_3_ and MoO_3_/WO_3_/rGO (**a**), equivalent circuit related to Nyquist plots (**b**) CV curves of MoO_3_/WO_3_ (**c**) and MoO_3_/WO_3_/rGO (**d**) in different scan rates.

To investigate the capability of MoO_3_/WO_3_/rGO (MWR) and MoO_3_/WO_3_ (MW) nanomaterials as supercapacitor electrodes in the field of energy storage, 2 M KOH solution was first prepared as an electrolyte, and the EIS analysis of both samples was performed in the frequency range of 1 mHz to 10 kHz at AC voltage of 0 V. Figure 3a shows the EIS analysis related to MW and MWR. The charge transfer resistance (Rct) for MW and MWR is respectively 33.76 and 20.25 Ω, which indicates the better electrical conductivity of MWR compared to MW. RGO causes a decrease in charge transfer resistance and also changes the angle of the Warburg line to a vertical state. The equivalent circuit related to Nyquist plots is drawn in Fig. 3b. In this figure R_s_ is solution resistance, R_ct_ is charge transfer resistance, CPE is constant phase element, and W is Warburg impedance.

Figures 3c and d are related to the CV analysis of MW and MWR nanocatalysts in 2 M KOH solution in the scan rate range of 20–100 mV/s. As seen in both figures, with an increase in the scan rate, we observed a growth in capacitive and Faradic currents. The CV curve corresponding to MWR is slightly wider than MW due to the presence of rGO in the matrix of MW. Considering that rGO has a capacitive behavior, it causes peak broadening and increases the capacitive current portion in CV analysis. Remarkably, the advantage of the synergism is that in addition to the increment of the electrical conductivity^28^ and electrochemically active surface area^29^, rGO also increases the current density in the microcellular structures, especially in the case of MWR compared to MW.

**Figure 4:**
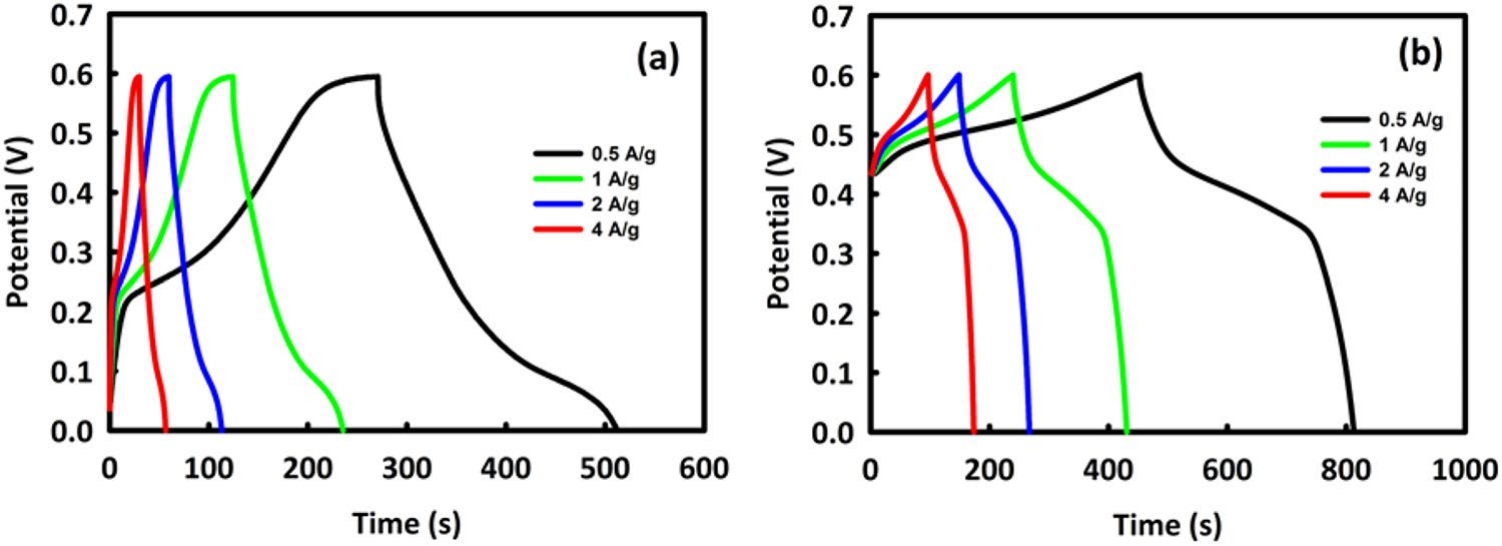
GCD analysis in different current densities for MoO_3_/WO_3_ (**a**) and MoO_3_/WO_3_ /rGO (**b**).

Galvanostatic charge/discharge (GCD) analysis was also carried out to check the capability of MW and MWR in energy storage at current densities of 0.5, 1, 2, and 4 A/g while again a 2 M KOH solution was used as the interaction medium. Figures 4a, b are the GCD analyses of MW and MWR, respectively. Using the equation C = it/m∆V (in this equation, i is the current density, t is the discharge time, m is the mass loading, and ∆V is the potential range)^30^, the specific capacitances of MW and MWR at a current density of 0.5 A/g were estimated as 452 and 583 F/g, respectively. The charging time for MW was 242 s and for MWR was 369 s, while the discharge times were 30 and 40 s, respectively.

**Figure 5:**
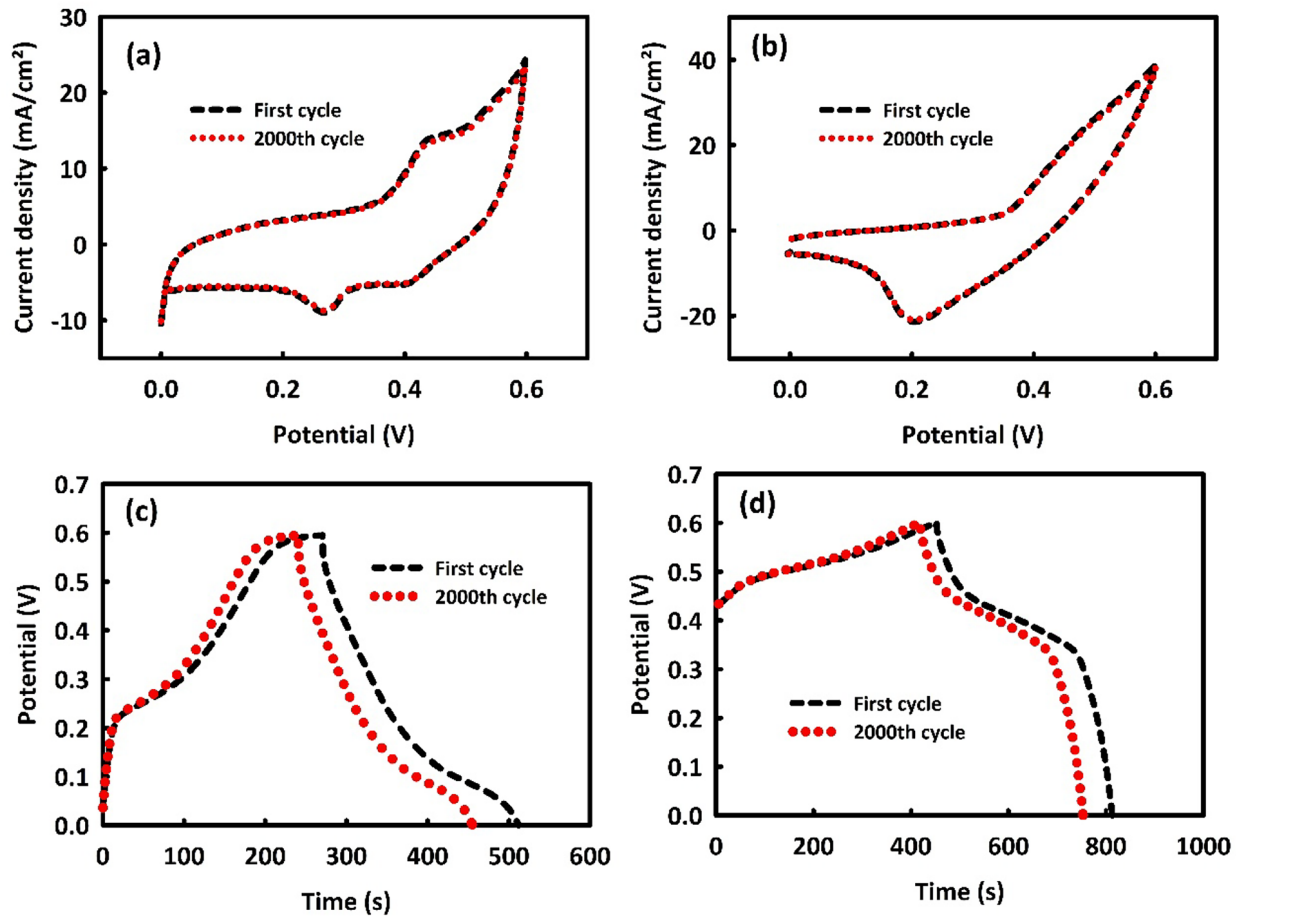
Cyclic stability after 2000 consecutive CV for MoO_3_/WO_3_ (**a**), MoO_3_/WO_3_/rGO (**b**) and Cyclic stability after 2000 consecutive GCD in current density of 0.5 A/g for MoO_3_/WO_3_ (**c**) and MoO_3_/WO_3_/rGO (**d**).

The stability of MW and MWR was determined by performing 2000 consecutive CV cycles in a 2 M KOH solution. Figure 5a, b show the stability of both mentioned materials, respectively, which is about 96.6% for MW and 97.9% for MWR.

Also, the stability of MW and MWR in GCD analysis was investigated by performing 2000 consecutive GCDs at a current density of 0.5 A/g. Furthermore, Fig. 5c, d belong to the investigation of MW and MWR stability. The stability values of these two electrode materials are 88.9 (MW) and 92.6% (MWR), which are relatively acceptable amounts for our proposed electrode materials.

Table 2 compares the performance of the MoO_3_/WO_3_/rGO electrode in energy storage as a supercapacitor electrode with similar electrodes that mostly have tungsten and molybdenum in their structure. As it is clear, the proposed electrode is a good and stable option in energy storage devices.

**Table 2:** Comparison of MoO_3_/WO_3_/rGO electrode efficiency with similar electrodes.

Electrode material	Electrolyte	Specific capacitance [F g^–1^]	Stability-cycle number	References
MoO_3_/WO_3_/rGO	2 M KOH	583	92.6%-2000 GCD	This work
SnWO_3_	1 M H_2_SO_4_	138	97.51%-2500 GCD	^31^
WO_3_/MWCNT||PANi	0.5 M H_2_SO_4_	120	80%-5000 GCD	^32^
WO_3_@NiCo_2_O_4_/rGO	6 M KOH	1211.75	91.51%-1000 GCD	^33^
WS_2_/WO_3_	2M KOH	570.1	87.4%-5000 GCD	^34^
NiMoO_4_/WO_3_/NF	3 M KOH	429.46	89.9%-10,000 GCD	^35^
WO_3_–MoO_3_ nanohybrid thin-films	1 M KOH	803.10	93.25%-5000 GCD	^36^
WO_3_–WS_2_/PPy	1 M Na_2_SO_4_	624	81%-5000 GCD	^37^
PANI/MoO_3_/h–BN	6 M KOH	518	Not reported	^38^
MoO_3_/g–CN	2 M KOH	852	Not reported	^39^
NiMoO_4_/MoO_3_	3 M KOH	3626	86%-2000 GCD	^40^
MoO_3_–Fe_2_O_3_ NC	1 M KOH	908	81.17%-2000 GCD	^41^
Ti_3_C_2_@MoO_3_	2 M KOH	624	84%-5000 GCD	^42^

#### MoO_3_/WO_3_/rGO and MoO_3_/WO_3_ nanocatalysts in the methanol oxidation reaction

**Figure 6:**
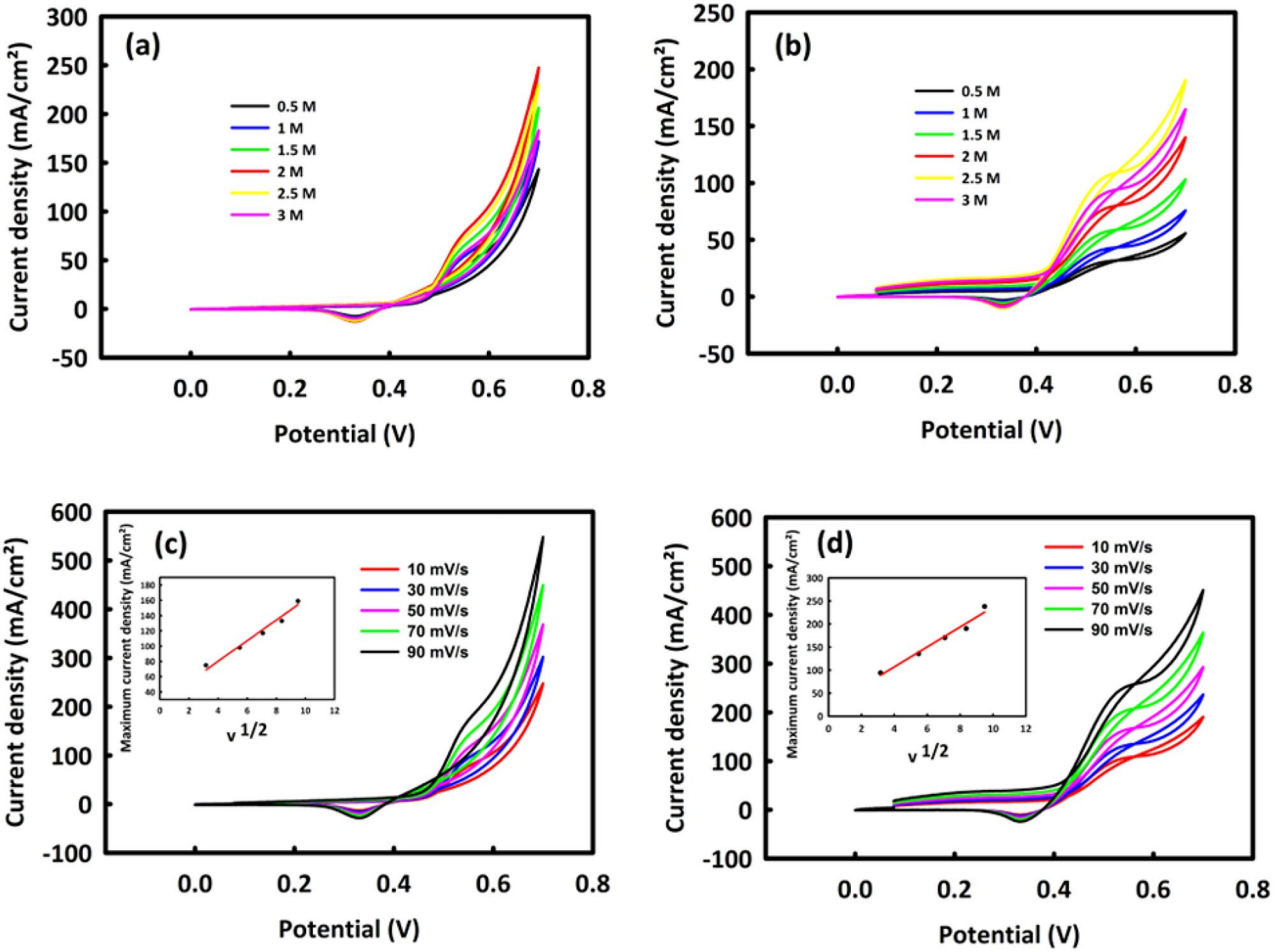
CV in different concentrations of methanol (0.5–3 M)/(2 M KOH) for MoO_3_/WO_3_ (**a**), MoO_3_/WO_3_/rGO (**b**) and CV in different scan rates in optimal concentrations of methanol /2M KOH for MoO_3_/WO_3_ (**c**), MoO_3_/WO_3_/rGO (**d**). The plot of maximum current density in term of square root of the scan rate for MoO_3_/WO_3_ and MoO_3_/WO_3_/rGO is shown in inset of figure (**c**) and (**d**).

The methanol oxidation process for MW and MWR nanocatalysts was evaluated by examining the behavior of these materials in different concentrations of methanol (0.5–3 M) when an alkaline medium (2 M KOH) was used and the scanning rate was set at 10 mV/s. As can be seen in the CV curve of Fig. 6a, MW has an oxidation peak at a potential of 0.7 V in the presence of methanol. At the methanol concentration of 2 M, we see a decrease in the current density of the oxidation peak. Regarding the behavior of MWR in the presence of methanol, it can be inferred that the peak of methanol oxidation indicates an overvoltage at 0.53 V, which is lower than MW (0.55 V), and also there is an upward trend in current density up to 2.5 M of methanol concentration (Fig. 6b). Seemingly, the surfaces of MW and MWR nanocatalysts are saturated at critical concentrations of methanol, and after these concentrations, methanol, and electrolyte penetrate very hardly into the core of the catalyst so that the MOR process cannot be carried out easily, and the oxidation current density increases with the increase in methanol concentration. The fact that the surface of the MWR catalyst is saturated with a higher concentration of methanol indicates a higher amount of electrochemically active surface of this nanocatalyst, which is the result of the presence of rGO inside its structure. As already pointed out rGO, not only expands the electrochemically active surface but also increases the electrical conductivity generally facilitating electron transfer as well as electrochemical processes.

We investigated also the behavior of MW and MWR nanocatalysts in 2 M KOH at different scan rates in the optimal concentration of methanol. It can be seen that by increasing the scan rate from 10 to 90 mV/s, the oxidation current density increases for both nanocatalysts. Figure 6c, d, belong to the behavior of MW and MWR at different scan rates, respectively, in the inset of which, the graphs of the maximum current density is shown against the square root of the scan rate. The linearity of these graphs with R^2^ = 0.995 for MW and R^2^ = 0.998 for MWR, indicates a diffusion-controlling mechanism for two nanocatalysts in the MOR process.

The proposed mechanism of methanol oxidation for two nanocatalysts can be explained as a 6-electron operation as follows^27^:

$$Catalyst + {\text{CH}}_{3} {\text{OH}} \to Catalyst - {\text{CH}}_{3} {\text{OH}}_{{ads}}$$

$$Catalyst - {\text{CH}}_{3} {\text{OH}}_{{ads}} + 4{\text{OH}}^{ - } \to Catalyst - \left( {{\text{CO}}} \right)_{{ads}} + 4{\text{H}}_{2} {\text{O}} + 4e^{ - }$$

$$Catalyst + {\text{OH}}^{ - } \to Catalyst - {\text{OH}}_{{ads}} + e^{ - }$$

$$Catalyst - {\text{CO}}_{{ads}} + Catalyst - {\text{OH}}_{{ads}} + {\text{OH}}^{ - } \to Catalyst + {\text{CO}}_{2} + {\text{H}}_{2} {\text{O}} + e^{ - }$$

**Figure 7:**
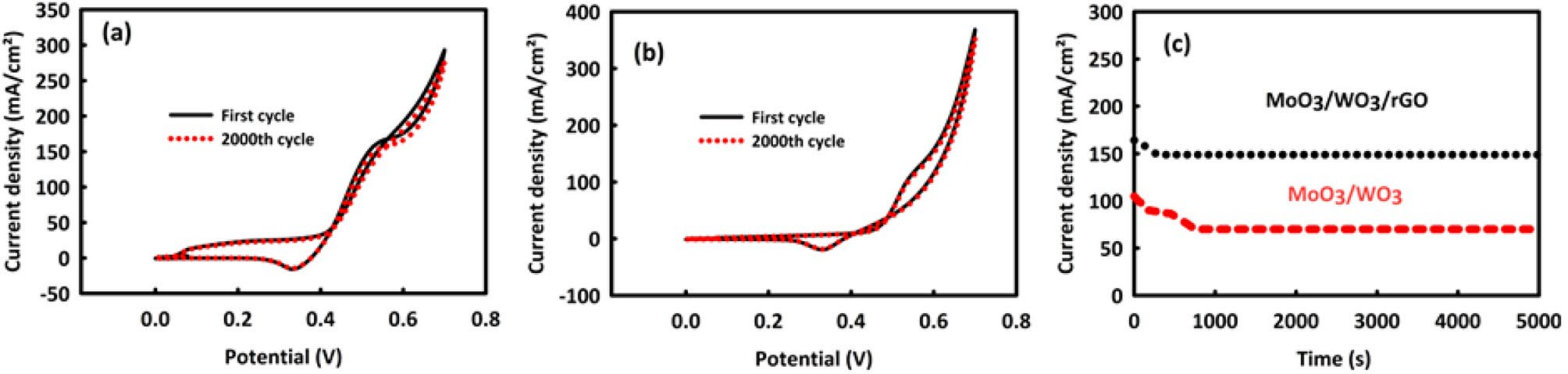
Cyclic stability after 2000 consecutive CV for MoO_3_/WO_3_ (**a**) and MoO_3_/WO_3_/rGO (**b**). Chronoamperometry analysis after 5000 s in peak potential and optimal concentration of methanol/2 M KOH for MoO_3_/WO_3_ and MoO_3_/WO_3_/rGO (**c**).

The stability values of MW and MWR nanocatalysts in the MOR process were evaluated by performing CV and Chronoamperometry analyses. Figure 7a, b are respectively related to 2000 consecutive CV cycles of MW and MWR in the optimal concentration of methanol into an alkaline environment (scan rate 50 mV/s). As it can be seen, after this number of consecutive CVs, MW stability is 92.8% and MWR stability is 94.4%, which are relatively good values. The Chronoamperometry analysis of MW and MWR in the oxidation peak potential was also carried out during 5000 s of nanocatalysts. Figure 7c is the chronoamperometry curve belonging to MW and MWR, in which the stability of this nanocatalyst is about 71% in the current density point of view. The stability of the current density for MWR in this analysis is 89%, which is much better than MW. Here, although both nanocatalysts are very stable, but MWR exhibits a more considerable stability aspect. The presence of rGO in the MW structure has increased the electrochemically active surface, and by increasing the contact surface between the electrolyte/methanol and the catalyst surface, an increase in the electrochemical stability of the catalyst is evident^43^.

#### MoO_3_/WO_3_/rGO and MoO_3_/WO_3_ nanocatalysts in the ethanol oxidation reaction

**Figure 8:**
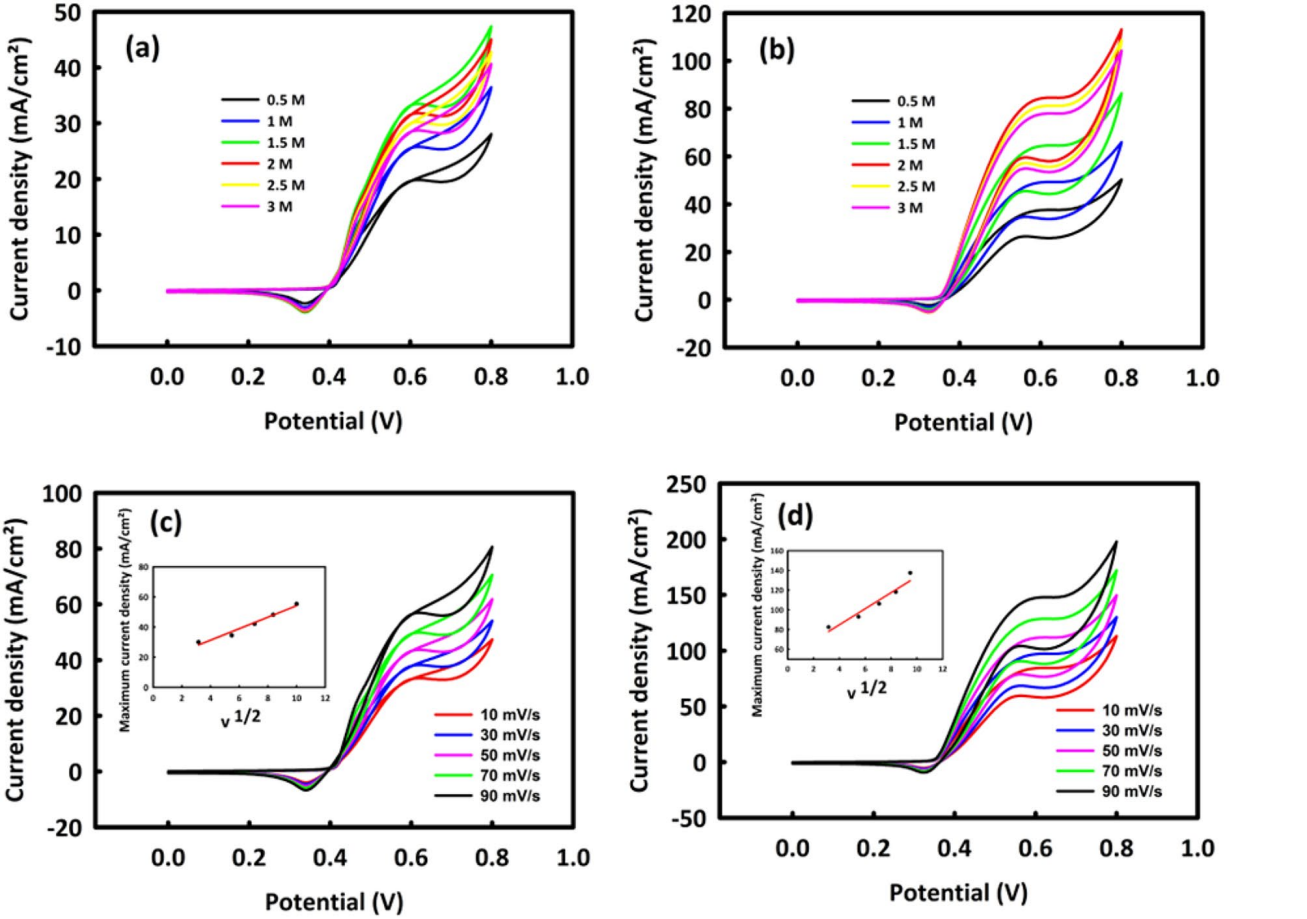
CV in different concentrations of ethanol (0.5–3 M)/(2M KOH) for MoO_3_/WO_3_ (**a**), MoO_3_/WO_3_/rGO (**b**) and CV in different scan rates in optimal concentrations of ethanol/2M KOH for MoO_3_/WO_3_ (**c**), MoO_3_/WO_3_/rGO (**d**). The plot of maximum current density in terms of the square root of the scan rate for MoO_3_/WO_3_ and MoO_3_/WO_3_/rGO is shown in the inset of figures (**c**) and (**d**).

Next, to investigate the capability of MW and MWR nanocatalysts in the ethanol oxidation process, different concentrations of ethanol (0.5–3 M) were added to a 2 M KOH solution as an electrolyte. In the CV analysis of MW and MWR nanocatalysts, which are illustrated in Fig. 8a, b, respectively, an oxidation peak is observed, the overvoltage of which is 0.59 V for MW and 0.58 V for MWR. It should be mentioned that CV analysis was performed in the potential range of 0–0.8 V at a scan rate of 10 mV/s. The critical concentration of ethanol for MW is 1.5 M and for MWR is 2 M, and suggestively from these concentrations onward, by saturating the surface of the catalysts, the penetration path of ethanol into the core of the catalysts is almost closed so that the anodic current density decreases^44^.

By performing CV analysis at different scan rates and drawing the square root of the scan rate according to the maximum current density, we studied the mechanism of the EOR process on MW and MWR. Figure 8c, d show the behavior of two nanocatalysts in the CV analysis. Oxidation current densities in both graphs have an upward trend with increasing the scan rate. The square root of the scan rate plotted against the maximum current density is present in the inset of each figure showing the diffusion-control mechanism in the EOR process on both nanocatalysts.

The ethanol oxidation mechanism on MW and MWR can also be suggested as follows^27^:

$${\text{Catalyst}} + {\text{OH}}^{ - } \to {\text{Catalyst}} - {\text{OH}}_{{ads}} + {\text{e}}^{ - }$$

$${\text{Catalyst}}+{{\text{CH}}}_{3}{{\text{CH}}}_{2}{\text{OH}}\to {\text{Catalyst}}-{{({\text{CH}}}_{3}{{\text{CH}}}_{2}{\text{OH}})}_{ads}$$

$${\text{Catalyst}} - ({\text{CH}}_{3} {\text{CH}}_{2} {\text{OH}})_{{ads}} + 3{\text{OH}}^{ - } \to {\text{Catalyst}} - ({\text{CH}}_{3} {\text{CO}})_{{ads}} + 3{\text{H}}_{2} {\text{O}} + 3{\text{e}}^{ - }$$

$${\text{Catalyst}}-{{({\text{CH}}}_{3}{\text{C}}O)}_{ads}+{\text{Catalyst}}-{{\text{OH}}}_{ads}\to {\text{Catalyst}}-{{({\text{CH}}}_{3}{\text{COOH}})}_{ads }+{\text{Catalyst}}$$

$${\text{Catalyst}} - ({\text{CH}}_{3} {\text{COOH}})_{{ads}} + {\text{OH}}^{ - } \to {\text{Catalyst}} + {\text{CH}}_{3} {\text{COO}}^{ - } + {\text{H}}_{2} {\text{O}}$$

**Figure 9:**
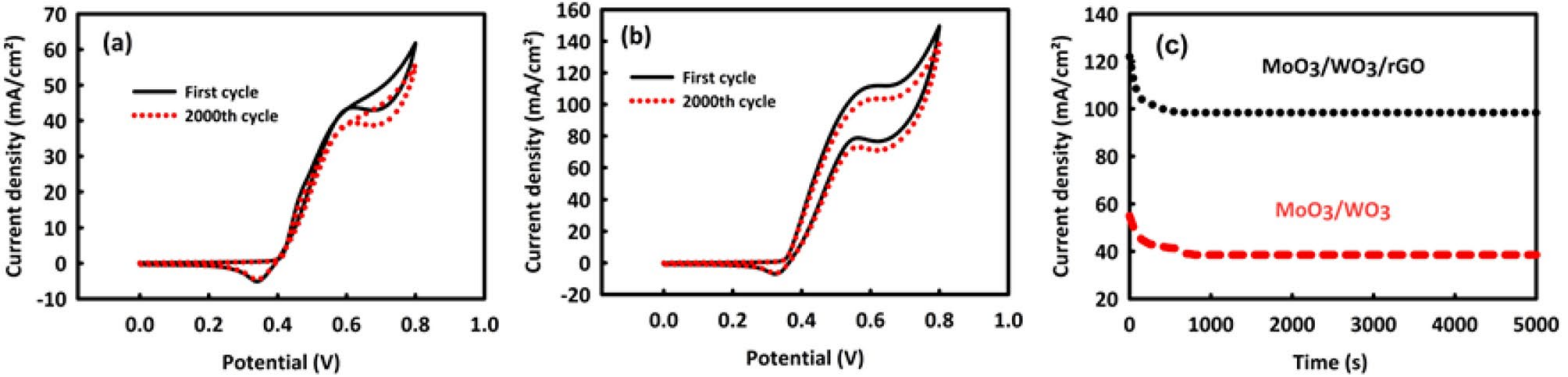
Cyclic stability after 2000 consecutive CV for MoO_3_/WO_3_ (**a**) and MoO_3_/WO_3_ /rGO (**b**) and chronoamperometry after 5000 s in peak potential and optimal concentration of ethanol/2 M KOH for MoO_3_/WO_3_ and MoO_3_/WO_3_ /rGO (**c**).

The results obtained from investigating the stability of MW and MWR nanocatalysts in the EOR process were relatively acceptable in 2000 consecutive CVs in the optimal concentration of ethanol at 50 mV/s of the scan rate. For MW and MWR (Fig. 9a, b), we see 90.1 and 92.6% of the stabilities in the anode current density, respectively. By performing the chronoamperometry test for 5000 s (at the oxidation peak potential) for MW and MWR nanocatalysts, shown in Fig. 9c, it can be found that these nanocatalysts have 70.7% and 82.1% stability in current density, respectively, which are reasonably acceptable values.

As it was mentioned, among the rare and expensive metals, platinum is the most efficient in the process of methanol and ethanol oxidation. Among other metals, nickel is considered a highly efficient catalyst for use in anodes of alcohol fuel cells due to its inherent electrocatalytic properties in various oxide and sulfide forms. Table 3 compares the efficiency of MoO_3_/WO_3_/rGO catalyst in MOR and EOR processes with other catalysts that are mostly based on nickel and platinum.

**Table 3:** MOR and EOR performances of MoO_3_/WO_3_/rGO with other researches.

Electrocatalyst	Electrolyte composition	Peak potential (V)	Current density (mA cm^–2^)	References
MoO_3_/WO_3_/rGO	2.5 M Methanol/2 M KOH	0.53	170	This work
MoO_3_/WO_3_/rGO	2 M Ethanol/2 M KOH	0.58	106	This work
Pt/Ni/SiO_2_–PANI	1 M Methanol/0.5 M KOH	0.24	144	^45^
NiCo/NiO–CoO/NPCC/GCE	0.5 M Methanol/0.5 M NaOH	0.32	178	^46^
Ni–Co/MOF/GCE	0.5 M Methanol/0.5 M NaOH	0.34	122	^46^
RGO/PANI/Pt/Cu	1 M Methanol/0.5 M KOH	0.27	60.41	^47^
Plastic derived Pt/C	3.0 M methanol/1 M NaOH	Onset potential (− 572 mV)	96.74	^48^
Commercial Pt/C	3.0 M methanol/1 M NaOH	Onset potential (− 603 mV)	130.81	^48^
ZrO_2_/NiO/rGO	0.7 M Methanol/0.5 M KOH	0.52	26.6	^49^
ZrO_2_/NiO/rGO	0.5 M Ethanol/0.5 M KOH	0.52	17.3	^49^
MnCo_2_O_4_/NiCo_2_O_4_/rGO	2 M Methanol/2 M KOH	0.58	24.76	^50^
ZnFe_2_O_4_–ZrO_2_/Pt	1 M Methanol/0.5 M KOH	0.35	104.74	^51^

## Conclusions

In the realm of energy storage and production including supercapacitors and alcohol fuel cells, the electrochemical stability and cheapness of catalysts and electrode materials are significantly important parameters. For this purpose, in this study, nanomaterials consisting of two widely used oxides, WO_3_ and MoO_3_, as well as the hybrid of these materials with reduced graphene oxide were fabricated. Ternary applications of these nanocatalysts as supercapacitor electrodes for energy storage, and as anodes for both methanol and ethanol fuel cells were probed. MWR with a capacitance of 583 F/g and cyclic stability of 92.6% in 2000 consecutive GCD cycles is considered a relatively brilliant option to be introduced as a novel material for supercapacitor electrode applications. The capability in the MOR and EOR processes is one of the other attractions of this nano-electrocatalyst. MWR with oxidation current density of 170 mA/cm^2^ at 0.53 V overvoltage and current density stability of 89% in chronoamperometry analysis during 5000 s in the MOR process, as well as oxidation current density of 106 mA/cm^2^ at 0.58 V and stability of 82.1% in chronoamperometry in EOR process, is a revealingly advantageous choice as an efficient and inexpensive nanocatalyst for alcohol fuel cell applications.

## Data Availability

Data are available on request from the corresponding authors (P.Salarizadeh and MB.Askari).
